# [Retracted] Glycyrrhetinic acid induces G1-phase cell cycle arrest in human non-small cell lung cancer cells through endoplasmic reticulum stress pathway

**DOI:** 10.3892/ijo.2026.5910

**Published:** 2026-07-06

**Authors:** Jie Zhu, Meijuan Chen, Ning Chen, Aizhen Ma, Chunyan Zhu, Ruolin Zhao, Miao Jiang, Jing Zhou, Lihong Ye, Haian Fu, Xu Zhang

Int J Oncol 46: 981-988, 2015; DOI: 10.3892/ijo.2015.2819

Following the publication of the above paper, it was drawn to the Editor's attention by a concerned reader that the control β-actin blots shown in Fig. 3A for the H460 cell line were strikingly similar to the β-actin blots shown in Fig. 2B for the A549 cell line. In addition, the p16 protein blots shown for the A549 cell line in Fig. 3A were strikingly similar to blots shown for the protein OPN in a paper published subsequently by different authors in the journal *Cell & Bioscience*. Finally, an independently performed software analysis of the data in this paper undertaken by the Editorial Office revealed that two protein bands positioned adjacent to each other in Fig. 3A (the Cyclin-D1 blots in the first two lanes for the H460 cell line) were potentially matching, suggesting that the data in this figure may have been handled inappropriately.

Given the problems identified with the assembly of data in Fig. 3, and the fact that some of the data in this paper have appeared elsewhere in the scientific literature, the Editor of *International Journal of Oncology* has decided that it should be retracted from the Journal on account of a lack of confidence in the presented data. The authors were asked for an explanation to account for these concerns, but the Editorial Office did not receive a reply. The Editor apologizes to the readership for any inconvenience caused.

